# Principles and Practice of Surgery

**Published:** 1886-11

**Authors:** 


THE PRINCIPLES AND PRACTICE OF SURGERY. *By* FRANK HASTINGS HAMILTON, A.M., M.D., LL.D. *Illustrated with 472 engravings on wood. Third Edition, revised and corrected. pp. 989. New York:* WILLIAM WOOD & CO., 1886. *Chicago:* W. T. KEENER.

In the preface to this edition, dated March 1st, 1886, the author disclaims any attempt to make the work an encyclopædia of surgery, and says that he describes in all the detail required by the practical surgeon, only those methods, which, in his experience, have been the most satisfactory, "or which, after careful consideration, most commend themselves." Since both anæsthetics and antiseptics have come into use during the professional life of the author, he claims the privilege "to institute comparisons, and to draw conclusions." This privilege he has exercised throughout the book as occasions are presented, and also in the concluding chapter on "The Art of

Primary Union," etc. He claims that the following conditions are necessary in order that primary union may occur, viz.:

1. A good, or at least average, state of the general health, and especially the absence of any systemic infection of dyscrasy.

2. The removal of all foreign bodies from the wound.

3. The effusion of coagulable lymph.

4. No unnecessary violence to the parts, from hands, sponges, sutures, adhesive strips or bandages.

The author claims that if the conditions mentioned above are not secured in incised wounds no amount of antiseptics can secure primary union. He does not believe that the use of antiseptics secures primary union by preventing the access of bacteria to the wound. He claims that Lawson Tait and Keith substantially agree with him, as do many other less distinguished men. The argument cannot be even summarized here, but it is clearly and forcibly put, and is entitled to fair and careful consideration by those who do not agree with the author.

The letter-press of the book is exceptionally good. As a practical guide in general surgery it is excellent, and it will stand, together with the lamented author's classical treatise on "Fractures and Dislocations," as a worthy record of a long, a useful and a distinguished professional life.

